# A Microfluidic Platform to Study Astrocyte Adhesion on Nanoporous Gold Thin Films

**DOI:** 10.3390/nano8070452

**Published:** 2018-06-21

**Authors:** Alexander E. Hampe, Zidong Li, Sunjay Sethi, Pamela J. Lein, Erkin Seker

**Affiliations:** 1 Department of Biomedical Engineering, University of California—Davis, Davis, CA 95616, USA; aehampe@ucdavis.edu (A.E.H.); zdli@ucdavis.edu (Z.L.); 2 Department of Molecular Biosciences, University of California—Davis, Davis, CA 95616, USA; sosethi@ucdavis.edu (S.S.); pjlein@ucdavis.edu (P.J.L.); 3 Department of Electrical and Computer Engineering, University of California—Davis, Davis, CA 95616, USA

**Keywords:** nanostructure, cell-material interaction, nanoporous gold, adhesion strength, astrocyte, focal adhesion, microfluidic flow-cell

## Abstract

Nanoporous gold (np-Au) electrode coatings have shown improved neural electrophysiological recording fidelity in vitro, in part due to reduced surface coverage by astrocytes. This reduction in astrocytic spreading has been attributed to the influence of electrode nanostructure on focal adhesion (FA) formation. This study describes the development and use of a microfluidic flow cell for imposing controllable hydrodynamic shear on astrocytes cultured on gold surfaces of different morphologies, in order to study the influence of nanostructure on astrocyte adhesion strength as a function of np-Au electrode morphology. Astrocyte detachment (a surrogate for adhesion strength) monotonically increased as feature size was reduced from planar surfaces to np-Au, demonstrating that adhesion strength is dependent on nanostructure. Putative mechanisms responsible for this nanostructure-driven detachment phenomenon are also discussed.

## 1. Introduction

Controlling cellular responses to implanted materials has long been an important focus in biomaterial design [1,2,3]. Structural modifications on the material surface influence the adsorption of extracellular matrix (ECM) proteins, which affects integrin-ligand binding and the formation of adhesive complexes [4]. This in turn regulates the spreading, growth, migration, and differentiation of adhesive cells [5]. The potential to control these functions without the use of chemical or pharmaceutical agents has motivated studies on cellular responses to material property modifications, namely substrate stiffness, surface chemistry, and topography at both the micro- and nano-scale [6,7]. 

Chronically implanted neural electrodes have significant potential when studying the brain and managing neurological disorders; however, adverse tissue responses, such as glial scar formation and meningeal encapsulation, remain significant problems [8]. Potential improvements to neural electrodes have been studied on many fronts, such as decreasing electrode form-factor [9], reducing stiffness [10,11], and depositing polymer coatings [12]. In addition, interfacial nanotopography has emerged as an important factor for influencing ECM protein layer formation and subsequently, cell adhesion [13]. In the context of neural electrodes, coatings that selectively inhibit adhesion of reactive cells while retaining neuronal proximity to the electrode, such as astrocytes, are highly desirable. This, in turn, should improve the durability and fidelity of the neural interface electrodes [14].

Nanoporous gold (np-Au) is a nanostructured material [15] suitable for a wide range of applications, from short nucleic acid sensing [16] to controlled drug delivery [17]. One important feature of np-Au is that its morphological features (e.g., pore width and ligament width) can be easily tuned by varying dealloying duration [18] or by thermal annealing [19]. Its nanostructure has also shown improvement in signal-to-noise in electrophysiological recordings [20]. As a possible explanation for enhanced recording fidelity, Chapman et al. have previously revealed reduced astrocytic coverage but unaffected neuronal coverage on np-Au surfaces compared to their planar gold (pl-Au) counterparts [21]. Furthermore, astrocyte focal adhesion (FA) contact area and focal adhesion number exhibited nanostructure-dependent changes [22], with an increase in focal adhesion number on np-Au films with smaller ligament widths and a drastic decrease in focal adhesion contact area on films with larger feature sizes. This suggests that different mechanisms guide focal adhesion formation on these nanostructure length scales. Since focal adhesion formation requires integrin organization via clustering with a critical size [23], the nanostructure may be inhibiting integrin clustering processes and consequently affecting adhesion strength. 

Using a facile microfabrication process in tandem with laser-annealed np-Au morphology libraries that present different electrode morphologies [19], we will report on the development of a microfluidic flow cell to study astrocyte adhesion strength on multiple nanostructured surfaces. We used shear-induced cell detachment as an indicator of adhesion strength [24]. We employed live cell imaging to keep track of the number of cells detached from the surfaces as a result of increasing hydrodynamic shear imposed by fluidic flow and quantified by a computational model. 

## 2. Materials and Methods 

### 2.1. Morphology Library Fabrication

The coating morphology libraries were prepared on polished 100 mm-diameter silicon wafers (University Wafer, South Boston, MA, USA), which were cut into thirds. A 1:4 solution (by volume) of hydrogen peroxide to sulfuric acid, called piranha solution, was used for sample cleaning. **Caution**: Piranha solution is corrosive and reactive with organic materials and must be handled with extreme care. Wafers were immersed in piranha solution for 5 min, then washed in deionized (DI) water and dried with nitrogen gas. Gold patterns were then deposited onto the chips via direct current sputtering (Kurt J. Lesker, Phillipsburg, NJ, USA) through a laser-cut polydimethylsiloxane (PDMS) film as the stencil mask. Two distinct masks were used: one consisted of 16 squares of size 2.6 mm × 2.6 mm, arranged in two rows (channels) by eight columns, while the other mask consisted of 8 rectangles of size 2.4 mm-width × 4.8 mm-height, arranged in a single row. The unstructured gold (pl-Au) libraries consisted of a chromium adhesion layer 160 nm-thick, with an overlaid 200 nm-thick gold layer. The preparation of np-Au libraries began by sputtering a 160 nm-thick chromium adhesion layer, followed by a 80 nm-thick gold corrosion barrier layer and a co-sputtered 600 nm-thick layer of gold and silver alloy (64% silver and 36% gold; atomic %). Gold-silver alloy samples were dealloyed by immersion in heated (55 °C) nitric acid (70%) for 15 min, resulting in the nanoporous morphology. Dealloyed samples were then kept in deionized (DI) water for one week, with a complete water change every 24 h. 

The chips with np-Au patterns were dried with nitrogen gas and annealed with a custom 532 nm continuous-wave laser, as previously reported [19]. A laser power of 600 mW at the surface was used in an alternating pattern with unannealed squares, yielding two sets of four similar coatings per microfluidic channel (to be described next). The annealing patterns for the two channels were mirrored to mitigate any discrepancies between the chips along the length of the channel. Characterization of each nanoporous morphology was performed using scanning electron microscopy (FEI Nova Nano-SEM430, FEI Company, Hillsboro, OR, USA). ImageJ, MATLAB, and Python scripts from previous studies were used in the analysis of feature sizes [19,25]. Ligament widths and pore areas are presented as a mean value plus or minus the standard error from three different image locations in three separate images. 

### 2.2. Flow Cell Preparation

Molds for microchannel fabrication were prepared from printable foil stickers (Silhouette, Lindon, UT, USA) [26]. The stickers were placed in 100 mm-diameter petri dishes, laser cut with a UV laser with parameters specified by an AutoCAD guide. After cutting, the sticker was peeled, resulting in a mold with raised channel regions measuring 3.5 mm × 40 mm for the first design (referred to as the *high-throughput* chip) and 3.5 mm × 22 mm for the second design (referred to as the *real-time* chip). Channel height was determined by measurements from both the petri dish and the PDMS mold surface using a 2D profilometer (Bruker Dektak XT, Billerica, MA, USA). 

PDMS microchannels were prepared from Sylgard 184, using a 1:10 ratio of curing agent to elastomer base. The mixture was poured into the petri dish mold and placed under vacuum for 1 h to remove air bubbles. The petri dishes were then placed on a hot plate at 80 °C for 2 h. The PDMS was peeled from the petri dish and cut into individual pieces with two channels per chip. Holes were punched at both ends of each channel using a 3 mm biopsy punch (Miltex, York, PA, USA). The morphology libraries were treated with oxygen plasma for 60 s on each side, followed by bonding the channels to the morphology libraries after plasma treatment for 30 s. After bonding, samples were placed under vacuum for 30 min, followed by 1 h incubation in cell culture media at 37 °C, with sterile glass cloning cylinders (Sigma, St. Louis, MO, USA) placed over channel inlets and outlets as media reservoirs. A small amount of silicone grease (Dow Corning, Barry, Vale of Glamorgan, UK) was applied around the base of each cloning cylinder to prevent liquid leakage.

### 2.3. Numerical Simulation of Shear Stress

**Figure A1:**
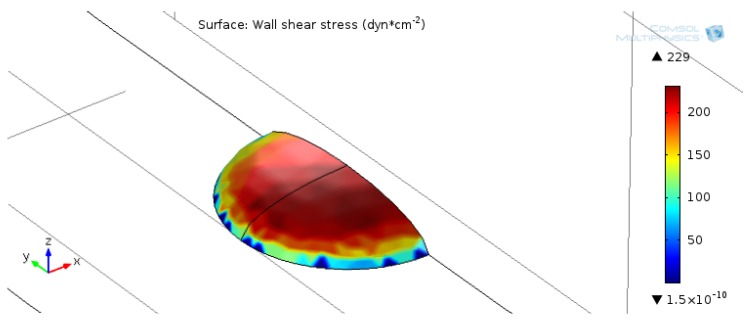
Simulation results for shear stress distribution across the cell membrane. The model assumes symmetry and simulates only half of the cell membrane. Shear stress (dyne/cm^2^) is plotted only on the cell membrane area and is maximized at the top of the dome. Cell dimensions: 18 µm radius, 5 µm height.

To relate volumetric flow rate with shear stress at the cell membrane, a multiphysics computational model (COMSOL, Burlington, MA, USA) was developed (Figure A1 in Appendix A). The simulation was based on a study using a similar model [27]. The simulation assumed laminar flow, which was realistic for the maximum experimental flow rate by calculation of the Reynolds number,

(1) $$\;Re=\frac{\rho vD_{h}}{\mu },$$

where ρ is flow medium density, v is the mean fluid velocity, $D_{h}$ is the characteristic or hydraulic diameter, and μ is the fluid dynamic viscosity. Values used were estimates: a density of 1000 kg/m^3^, a velocity of 0.34 m^3^/s, and a characteristic diameter equal to twice the channel height, or 280 µm, were assumed. The Reynolds number did not exceed 100 for these calculations, indicating laminar flow conditions. The entrance length was estimated similarly as

(2) $$\;L_{h}=0.05ReD_{h}$$

Using the same hydraulic diameter and a Reynolds number of 100, the entrance length did not exceed 1.4 mm, which was below the minimum length from the channel entrance/exit to the gold sample, at approximately 5 mm. The simulation volume was 140 µm high, 300 µm long, and 50 µm wide. Walls with an applied no-slip boundary condition were defined at the top and bottom of the channel. The inlet velocity was set to 0.33 m/s, which was the average linear velocity for a flow rate of 10 mL/min in a 0.14 mm × 3.5 mm cross-section. Symmetry was assumed for both sides of the simulation. The cell shape was approximated by a dome shape, with a radius of 18 µm and a height of 5 µm. The radius was estimated by averaging actin immunofluorescence and normalizing to the cell count. The chosen height was consistent with measurements of primary astrocyte cell heights [28]. By the symmetry specification, only half of the cell was included in the simulation. Shear stress over the surface of the cell was calculated as shear rate multiplied by dynamic viscosity. The model assumed that the channel geometry would not deform due to pressure.

### 2.4. Astrocyte Cell Culture

Primary rat cortical cells were obtained from day 0 perinatal Sprague-Dawley rats (Charles River Laboratories, Hollister, CA, USA), following procedures described elsewhere [29,30]. All studies were conducted according to protocols approved by the Institutional Animal Care and Use Committee of the University of California, Davis. Dissociated cortical cells were plated initially in T75 flasks (Corning, NY, USA) at a density of 40,000 cells/cm^2^ in growth media (Dulbecco’s Modified Eagle’s Medium (DMEM) + l-glutamine and sodium pyruvate, 10% heat inactivated fetal bovine serum, 1% penicillin/streptomycin (Invitrogen, Carlsbad, CA, USA). Cells were incubated at 37 °C in 5% CO_2_ for at least 7 d, with a complete medium change after the first 24 h and every 4 d thereafter. After sufficient time to expand, astrocytes were isolated following previously established protocols, which are known to produce 98% pure astrocyte cultures [31,32]. Briefly, cytosine β-d-arabinofuranoside (Ara-C, a mitotic inhibitor purchased from Sigma-Aldrich) was added to the flasks at a final concentration of 1 μM to suppress fast-dividing cells such as fibroblasts and microglia, as well as to prevent astrocytes from balling up and detaching during cell division. The flasks were moved to an orbital shaker to remove unwanted cell types (e.g., microglia) and were left overnight (≥6 h) at a setting of approximately 70 rpm, with total media replacement after removal from the shaker. Cells were then given at least 2 d to allow for adhesions to return to normal before seeding them into microfluidic devices. 

To prepare for seeding, cells in T75 flasks were first washed in phosphate buffered saline (PBS) without calcium, trypsinized for 2–5 min, and then transferred to a centrifuge tube with added culture media. Tubes were centrifuged, supernatant was discarded, and cells were resuspended at a density of approximately 750,000 cells/mL. Media was aspirated from reservoirs at either end of the device, and the suspension was introduced into the reservoir at one end. Chips were placed in the incubator and cells were given 20 min to settle before removing the suspension from both reservoirs. Media was then replenished every 2 h by removing media from reservoirs and filling one reservoir with 450 μL fresh media. 

### 2.5. Cell Imaging

After approximately 4 h, cells were prepared for live counting. One reservoir was emptied, and the opposite reservoir was filled with media to 450 μL, with one drop of NucBlue Reagent (Invitrogen, Carlsbad, CA, USA) added. Chips were incubated for 15 min before cloning cylinders were removed and inlet and outlet holes were temporarily plugged with sealed silicone tubing. Chips were imaged with an inverted fluorescent microscope (Zeiss, Oberkochen, Germany). Depending on chip design, samples were used in high-throughput experiments or real-time experiments as detailed below. In both experiments, cells were sheared after 6 h in culture. 

**Figure A2:**
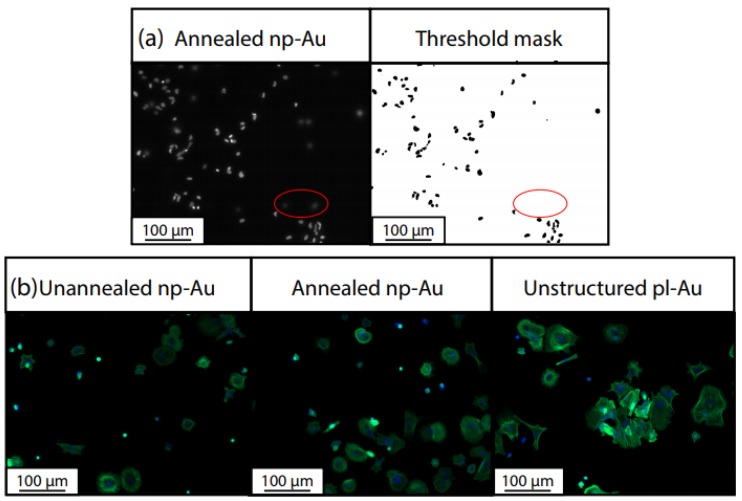
Representative images of pre- and post-experiment cell counting. (**a**) Pre-experiment image of cells seeded for 4 h, stained with NucBlue Live Reagent, with threshold mask shown to the right. The threshold method was chosen to minimize the number of (dim, low-contrast) floating cells in the count. An example of out-of-plane cells absent from the thresholded image are circled in red. (**b**) Fluorescence microscopy images of fixed cells, showing nuclei (DAPI) in blue and f-actin (phalloidin) in green. Thresholds were computed only for the DAPI channel.

The high-throughput experiment was divided into two groups: *shear* and *no-shear*. Cultures were sheared with warmed (37 °C) PBS with calcium and magnesium, delivered via a syringe pump (Harvard Apparatus, Holliston, MA, USA) set to infuse mode. Flow rate was set at 10 mL/min for a duration of 2 min. Cells were immediately fixed using 4% paraformaldehyde in PBS (Affymetrix, Santa Clara, CA, USA). The no-shear control group, to which no flow was delivered, was fixed after gently washing with PBS with calcium and magnesium, delivered via gravity-driven flow. The PDMS layer was peeled with a razor blade before staining. Cells were stained using Alexa Fluor-conjugated phalloidin (1:500) for cytoskeletal visualization. Samples were also counterstained with 4′,6-diamidino-2-phenylindole (DAPI) to quantify cell number (Figure A2). All antibodies were purchased from ThermoFisher Scientific. 

Images were taken at 5x magnification in the same approximate areas before and after shearing for the high-throughput experiment. Overlapping images were stitched with the ImageJ stitching plugin [33]. Stitched images were cropped to equal sizes before thresholding. The Otsu threshold method was chosen for pre-experiment cell counts, whereas the Triangle threshold method was chosen for post-experiment cell counts. Cellular detachment was quantified by

(3) $$\;\frac{x_{0}-x_{f}}{x_{0}},$$

where $x_{0}$ is the pre-experiment count and $x_{f}$ is the post-experiment count (Figure A2).

For the real-time experiment, chips were removed from the incubator, reservoirs were detached, and channel entrances and exits were plugged except for the channel to be sheared. Tubing was connected and chips were placed upside-down onto the microscope stage. Cultures were sheared with warmed (37 °C) PBS with calcium and magnesium, delivered via a programmable syringe pump (World Precision Instruments, Sarasota, FL, USA) set to infuse mode. Before starting real-time image acquisition, the pump was set to infuse at 0.1 mL/min for roughly one min to remove floating cells from the imaging area. A flow rate regimen consisting of 30 s of flow at successive rates of 2, 4, 6, and 8 mL/min were performed, followed by 60 s of flow at 10 mL/min. One area of the gold surface was imaged at 5× magnification exactly every 5 s. Cell counts in these regions ranged from 15 to 80 cells at the start of the experiment. Trials were carried out sequentially by successive channels, then by chips.

Studies involving morphology libraries were performed with an internal sample size of four repeats per np-Au morphology. All reported values are averages with error bars corresponding to the standard deviation of each averaged data set unless otherwise noted. A two-tailed Student’s *t*-test assuming unequal variance was used to identify differences between two different sample groups. Statistical significance was determined by *p*-values below 0.05.

## 3. Results and Discussion

### 3.1. Device Fabrication

**Figure 1:**
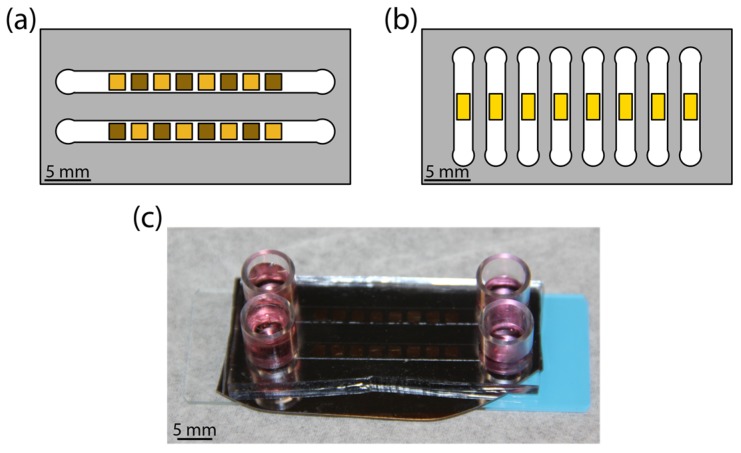
Illustration of the microfluidic flow cell. (**a**) Colorized schematic of high-throughput chip with nanoporous gold (np-Au) morphology library. Gold color patterns reflect the extent of annealing, where lighter squares represent annealed np-Au and darker squares representing unannealed np-Au. (**b**) Colorized schematic of real-time experiment chip with planar gold (pl-Au) substrate. (**c**) Photograph of a representative assembled np-Au chip with culture media in reservoirs.

The devices consisting of microchannel-encapsulated thin film coatings allowed for a controlled study of the influence of fluidic shear and coating morphology on astrocyte attachment. Schematics of the platform are shown for the high-throughput chip (Figure 1a) and for the real-time chip (Figure 1b). Each morphology library chip was composed of either all pl-Au or all np-Au. For np-Au, an alternating pattern of annealed and unannealed squares was obtained by in situ laser annealing. The sample areas were chosen to accommodate a sufficient population of cells (order of magnitude 100) while fitting within the microchannel. Media replenishment was provided via reservoirs at channel entrances and exits (Figure 1c). 

The microchannel height was obtained by profilometer measurements and was 138.0 µm on average, with a standard deviation of 0.3 µm. Channel lengths were 40 mm for the high-throughput chip and 22 mm for the live chip. All channel widths were 3.5 mm. The maximum shear stress obtainable by syringe pump-driven flow was nominally 200 dyne/cm^2^, which was lower than reported shear stresses required to detach 50% of well-spread cells, but was within the range required to detach cells seeded on micropatterned surfaces [34]. Our simulation (Figure A1) provided an estimate of the shear stress that an average astrocyte experiences, although we observed variations in spreading on all surfaces (Figure A2). 

**Figure 2:**
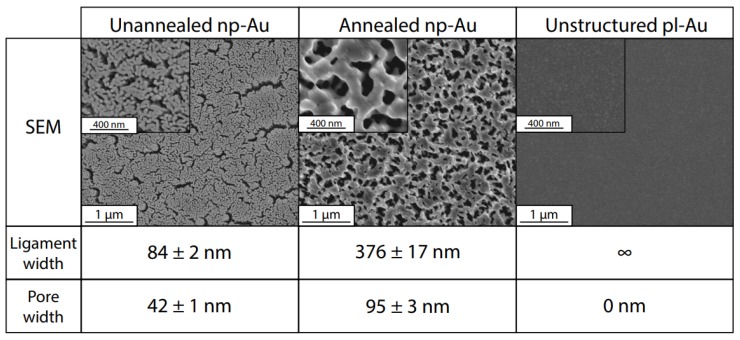
Comparison of morphology of annealed and unannealed nanoporous gold (np-Au) and planar gold (pl-Au). Scanning electron microscopy (SEM) images are shown at 50,000× magnification (inset: 100,000× magnification). Ligament and pore widths are reported as averages ± standard error.

Photo-thermal annealing resulted in np-Au coarsening due to enhanced surface diffusion of gold atoms and smoothing of gold ligaments with small radius of curvature, as described in detail elsewhere [19]. Figure 2 presents each substrate in increasing order of ligament width. The planar gold control group modeled a surface of infinite ligament width and zero pore width. Laser annealing caused a substantial increase in both ligament and pore width, increasing to 376 ± 17 nm from 84 ± 2 nm in ligament width and to 95 ± 3 from 42 ± 1 in pore width, compared to the unannealed sample. The ligament width of unannealed np-Au is larger than a previously computed value (30 nm) from our group [22], primarily due to the wider ligaments near surface cracks in the current study (Figure 2).

### 3.2. Influence of Coating Morphology on Adhesion

**Figure 3:**
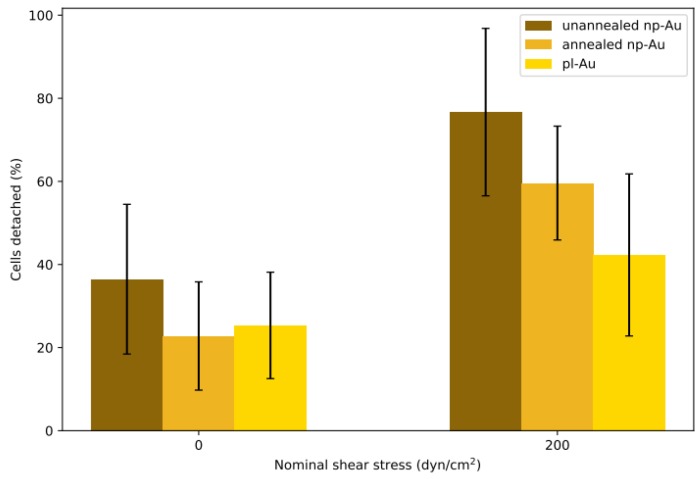
Detachment results for batch experiments. Two min of hydrodynamic shear flow detached cells seeded in the channels for 6 h. The no-shear control group at 0 dyne/cm^2^ was only gently washed with phosphate buffered saline (PBS) before fixation. Percent detachments are reported as averages ± one standard deviation. Sample sizes (n) for each group, from left to right: 24, 24, 47, 11, 7, 28 coating patterns.

Each flow cell was subjected to either *shear* or *no-shear* conditions after 6 h incubation, which provided sufficient but not permanent attachment that was essential for conducting the detachment study with respect to varying shear stress. A previous study by Gallant et al. reported that NIH3T3 fibroblasts reached steady-state adhesion after about 4 h [34]. While the 6 h incubation duration in this study is longer, purified primary astrocytes, which are not as homogeneous as NIH3T3 fibroblasts, may require a longer duration to reach full adhesion strength. The percent detachment (difference in pre- and post-experiment counts normalized to the initial cell count) at each condition is reported in Figure 3. Each morphology pair was statistically different (*p* < 0.05 by Student’s *t*-test) when the no-shear and shear groups were compared. While each morphology was different within the shear group, only unannealed np-Au exhibited a statistically significant difference compared to all other morphologies in the no-shear group. In the no-shear control group, there were between 20% to 40% fewer cells in the counts after fixation, suggesting that the initial estimate included some non-adhered cells which were washed away in the fixation step. The detachment on unannealed np-Au was statistically significant (*p* < 0.05) compared to the other two morphologies at no-shear. In the shear group, detachment appeared to decrease as ligament width increased: the unannealed np-Au group experienced the greatest detachment at 76%, for annealed np-Au, an intermediate detachment level of 59%, and for pl-Au, the lowest detachment at 42%. Taking the difference between the shear and no-shear groups, the adjusted detachment for both np-Au morphologies was approximately 40%. For pl-Au, the difference was lower at roughly 15%. The general trend of reduced cellular attachment with decreasing ligament width was consistent with what has been observed previously for reduced cell coverage on np-Au surfaces [35], suggesting that adhesion strength plays a role in cellular spreading.

**Figure 4:**
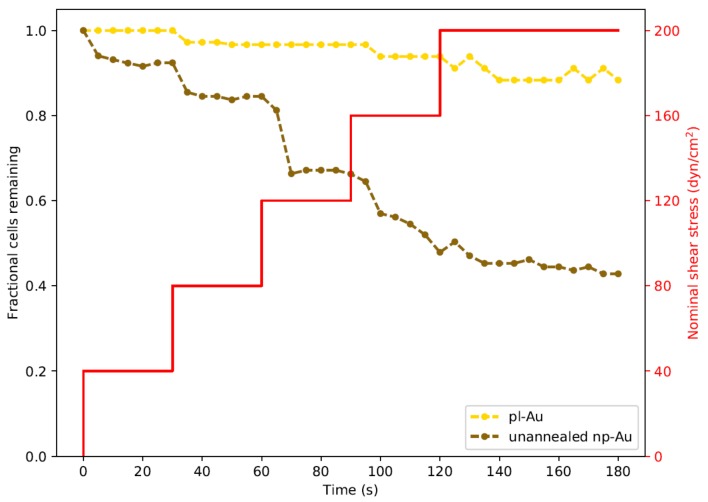
Real-time monitoring of cell detachment (surrogate for adhesion strength) as a function of increasing shear stress. Cells were subjected to increasing levels of shear stress as shown by the red staircase curve. Images were taken every 5 s. Two trials were performed for each morphology and were plotted as averages. Each data point for fractional cells remaining was normalized to the cell count at the start of each experiment.

In order to study the influence of shear stresses that lay between the two extremes illustrated in Figure 3, we employed a real-time imaging approach to monitor cell detachment. We specifically focused on unannealed np-Au and pl-Au, since they exhibited the largest difference in detachment (Figure 3). By increasing flow rate with a step-wise manner in 30 s intervals, we obtained a range of shear stresses and tracked detachment over a three min-long duration (Figure 4). With increasing flow rate (hence shear stress), there was a gradual increase in the number detached cells. Consistent with what has been observed earlier, more cells detached from the np-Au coating. The gradual increase in cell detachment highlights that while np-Au overall hinders cellular adhesion, there is a distribution of different cell adhesion strengths.

### 3.3. Influence of Focal Adhesions on Adhesion Strength

It has been reported that focal adhesion assembly accounts for approximately 30% of total cellular adhesive strength [34]. The remaining percentage was attributed to intracellular integrin-actin binding and clustering mechanisms. In addition, the same study showed that stable adhesion requires only a small fraction of the total available adhesive area, as adhesion strength maximizes before adhesive area allows for complete spreading. Furthermore, different integrin subtypes are known to contribute to adhesion strength. Integrin α_5_β_1_, for example, has been implicated in adhesion strengthening, whereas integrin α_v_β_3_ is responsible for mechanotransduction and does not significantly contribute to adhesion strength [36]. A fibronectin patterning study showed that there is a nanoscale area threshold for mature focal adhesion formation [37], which is dependent on cytoskeletal tension. The size threshold was determined to be between 250 × 250 nm and 333 × 333 nm, which lies between the average length scale (i.e., ligament width) for the annealed and unannealed np-Au films used in this study. Also, a critical RGD peptide spacing (corresponding to pore width in this study) of 58 nm is required for focal adhesion formation [38]. Larger ligand spacings decrease stability of focal adhesion formation and reduce spreading substantially. While we attributed the differences in astrocyte detachment (adhesion strength) mainly to the substrate nanotopography, it is important to mention that other factors such as surface chemistry and mechanical stiffness can play an important role in dictating cellular adhesion strength [39]. In addition, as described in our previous study, for short incubation durations, the probability of cells forming focal adhesion to a pitted surface upon reaching the surface may play a role [35].

**Figure 5:**
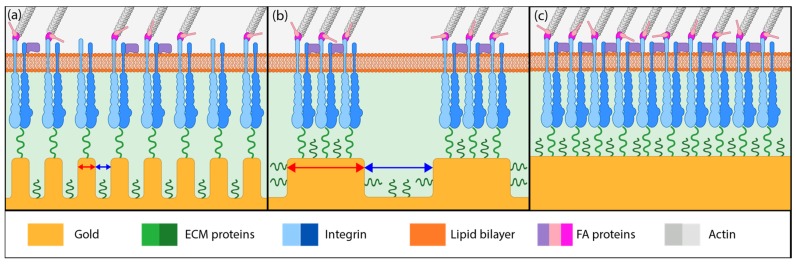
Illustration of different putative mechanisms of cellular adhesion onto coatings of various morphology. (**a**) On unannealed np-Au, integrin clusters may bridge multiple ligaments due to smaller pore widths but adhesive complexes cannot mature due to small ligament width (red arrows). (**b**) On annealed np-Au, integrin clustering is limited by the larger pore widths (blue arrows), limiting focal adhesion (FA) formation to individual ligaments. (**c**) On planar gold, FA maturation is uninhibited and FA size is large.

Based on the literature reports and the cell detachment (adhesion strength) data (Figure 3 and Figure 4), we propose that nanostructure (i.e., ligament width and pore width) influences both focal adhesion formation and integrin clustering (Figure 5), thus dictating the overall adhesion strength observed. It is plausible that different mechanisms play a dominant role with respect to the length scales embodied in the different np-Au morphologies. For pl-Au, that provides a continuous smooth surface for cell spreading, no restriction on focal adhesion formation (Figure 5c), and the least cellular detachment (highest adhesion strength) was observed (Figure 3 and Figure 4). For annealed np-Au, as ligaments thickened in the annealing process, the pore width increased accordingly (Figure 2). The mean spacing between the ligaments (95 nm) therefore became too large to allow for FA formation across pores (58 nm), as illustrated in Figure 5b. However, the larger ligament width (376 nm) was above the threshold for individual focal adhesion formation (250 nm), thus stable focal adhesions could form. For unannealed np-Au, while the mean pore width (42 nm) permit FA assembly across several ligaments (Figure 5a), the ligament width (84 nm) was below the critical threshold for FA formation. The substantial loss of adhesion strength would therefore be due to a decrease in integrin cluster density, leading to numerous nascent FAs which cannot mature. Without sufficiently high generation of traction force, cytoskeletal tension on these nascent FAs would lead to destabilization and the observed high levels of cell detachment. It is important to end the discussion by stating that the putative mechanisms described in Figure 5 would plausibly be most prominent for cells reaching a steady-state or close-to-steady-state adhesion strength. However, the cell adhesion behavior following short attachment duration was still significantly dependent on the coating morphology as shown in Figure 3 and Figure 4. It is expected that the behavior in this transitional cell attachment regime was driven by a combination of the aforementioned FA formation and integrin clustering mechanisms as well as the kinetics of FA establishment on the complex nanotopography (composed of ligaments and voids) [35].

## 4. Conclusions

We have shown that nanoporous gold morphologies reduce cell adhesion strength relative to planar surfaces, as judged by higher levels of cell detachment under fluidic shear stress. We attributed the differences in cell adhesion to an interplay between integrin clusters ability to span pore widths and to mature on ligaments of a critical width, both of which are differentially pronounced in fine and coarse np-Au coating morphologies, respectively. In addition, we noted that in this transitional cell attachment regime (close-to-steady-state cell adhesion state), the kinetics of focal adhesion formation on the complex nanotopography also played a role. The flow cell design coupled with the coating morphology libraries allowed for high-throughput investigation of morphology and shear stress on cell adhesion. The issue of FA destabilization due to cytoskeletal tension could be further explored by comparing wild-type cells with mutants expressing vinculin or talin head domains [37], which drive integrin-ligand clustering without linking the adhesive complex to the cytoskeleton. Further studies should provide insight into mechanotransduction events invoked at the gene level using RNA-seq of the astrocytes cultured on different coating morphologies.
